# Severe Constipation, Fecalith, and Giant Fecaloma in a Patient With Severe Intellectual Disabilities: A Case Report

**DOI:** 10.7759/cureus.79099

**Published:** 2025-02-16

**Authors:** Matthew Baer, Alec K Donohue, Andrew Anklowitz, Dana Poloni, CJ Quach

**Affiliations:** 1 Internal Medicine, Mount Sinai Morningside, New York, USA; 2 General Surgery, Womack Army Medical Center, Fort Liberty, USA; 3 General Surgery, Eisenhower Army Medical Center, Fort Eisenhower, USA

**Keywords:** individualized management strategies, intellectual and developmental disability, mechanical large bowel obstruction, multi-disciplinary teams, refractory constipation

## Abstract

Medical and surgical treatment in patients with intellectual disabilities (IDs) presents a challenge to physicians and surgeons alike, as informed consent and longitudinal treatment are difficult to achieve. Severe constipation has a high prevalence in persons with IDs and is found in this population at a rate almost double that experienced by the general population.

This case report highlights a middle-aged male with severe IDs, who presented with abdominal distention and behavior consistent with abdominal pain from constipation. The patient was found to have severe, chronic constipation, with an associated large, calcified fecalith in the rectum and an impressively expanded sigmoid and descending colon, secondary to a massive, chronic fecaloma. Resolution of the cause of constipation could not be achieved due to a combination of social factors, thereby highlighting the importance of advocating for and developing long-term treatment plans for prevention in such patients.

## Introduction

The American Association on Intellectual and Developmental Disabilities defines intellectual disability (ID) as “a condition characterized by significant limitations in both intellectual functioning and adaptive behavior, the collection of conceptual, social, and practical skills that are learned and performed by people in their everyday lives, and that originates before the age of 22” [1]. Intelligence quotient (IQ) criteria for patients with profound and severe IDs range from <20 to 20-40, respectively, and also include an inability to complete everyday tasks required for independent functioning [2].

Constipation is a significant health issue for individuals with IDs, affecting 25%-70% of this population [3,4]. Risk factors include severe IDs, cerebral palsy, limited mobility, dysphagia, and polypharmacy, particularly antipsychotics and antiseizure medications [5-8]. The condition is often underdiagnosed due to communication barriers and can lead to serious complications, including behavioral disturbances, intestinal obstruction, and even death [9-11]. Management primarily relies on laxatives, despite limited effectiveness. Alternative approaches, such as dietary fiber intake and abdominal massage, show promise but require further research [3,9,10]. Improved bowel health monitoring, medication reduction, and individualized care plans are recommended [6,9]. Given the high prevalence and potential severity, constipation should be actively considered as a diagnosis in this population [3,11].

Many patients with IDs experience communication challenges [12]. As such, communication acts as a barrier in this population when expressing discomfort or understanding the need for regular evacuation of their bowels. Delayed response to bowel urges or inadequate efforts to evacuate bowel contents may result from these factors. Moreover, some individuals with IDs may have limited dietary choices or difficulty in managing their diet, leading to insufficient fiber intake or inadequate hydration, both of which play critical roles in maintaining regular bowel movements [6,9,13]. Combined, these factors contribute to the increased prevalence of severe constipation in this population [3]. Addressing these issues often requires a multidisciplinary approach, including primary care providers, allied health professionals, medical subspecialists, and behavioral health specialists to improve bowel management and overall quality of life for individuals with IDs [9,12-14].

The standard of care for constipation in patients with IDs involves a comprehensive and individualized approach to address their unique challenges. Healthcare providers should conduct a thorough assessment of the patient's medical history, cognitive abilities, mobility, and communication skills to tailor the management plan accordingly [13,14]. Treatment typically includes dietary modifications, such as an increased fiber intake under the supervision of a qualified physician, and water intake, as well as scheduled toileting routines to establish regular bowel habits. Laxatives or stool softeners are commonly prescribed to promote bowel movements [6,9,14]. Caregiver education is essential to ensure understanding and cooperation in managing constipation effectively. Continuous monitoring and follow-up are essential to assess progress and prevent complications [10,13-15].

Once lifestyle changes and medical management, including oral bowel regimens, decompression per rectum, and trans-anal irrigation, have been exhausted, surgery may be the last chance for these patients to experience an improvement in their quality of life [16,17]. Given that the state has a legal charge over many patients in this population, the road to surgery can be wrought with many obstacles, including the informed consent process [18].

## Case presentation

This case report highlights a 52-year-old male with a history of profound IDs and chronic constipation. This patient lived in a group home and was brought in by a home assistant, as the patient was non-verbal at baseline. The assistant reported that he was eating and drinking normally and taking his daily MiraLAX; however, he was unable to have a bowel movement over the last two weeks. Prior to the presentation, the patient was reported as having small, daily bowel movements, continued to pass flatus, and had no reported history of needing mechanical fecal disimpaction. Physical examination revealed an agitated patient with a non-tender but severely distended abdomen, with active bowel sounds.

**Figure 1:**
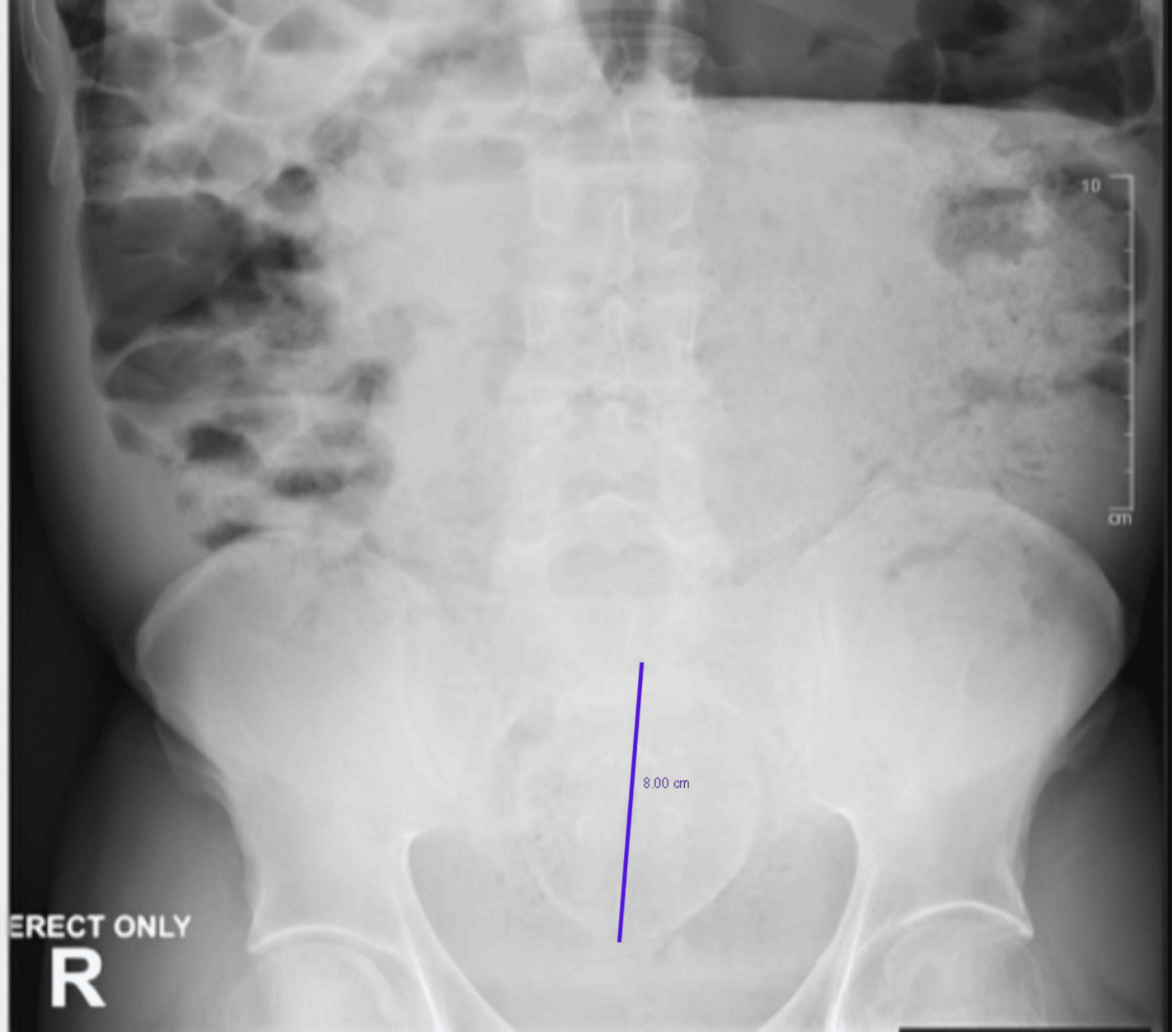
Abdominal X-ray showing a calcified fecalith measuring up to 8 cm in diameter.

Prior to obtaining imaging, chart review showed limited workup for chronic constipation, apart from X-rays from previous emergency room admissions dating back 13 years. At the time, the patient had a state-appointed healthcare proxy; however, no previous documentation about long-term goals of care from the proxy was found. As per the caregiver and chart review, the patient had never been seen in consultation by gastroenterology or a general/colorectal surgical team. Prior imaging was scarce, aside from several abdominal X-rays, which showed evidence of an 8.0 cm fecalith starting to form five years ago (Figure 1).

**Figure 2:**
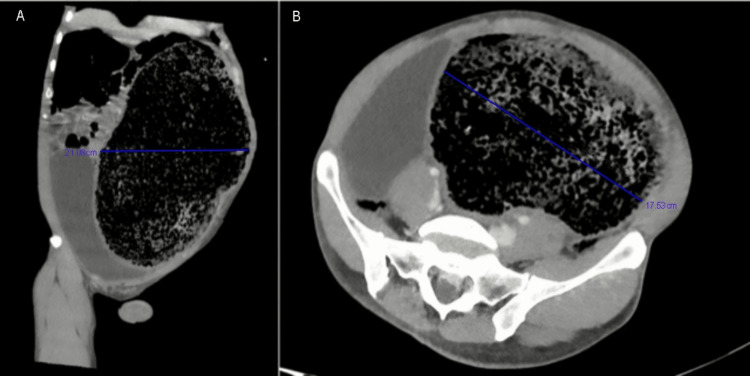
CT abdomen and pelvis with IV contrast depicting massive fecaloma. Image A highlights a fecaloma measuring 21.08 cm anterior to posterior. Image B demonstrates a transverse fecaloma measurement of 17.53 cm.

The patient was given senna glycosides, polyethylene glycol, and a tap water enema; manual disimpaction without general anesthesia was performed, with only minimal bowel movement elicited. He was subsequently taken for CT imaging, which showed an extremely large stool burden with a significant mass effect on the internal organs; the sigmoid colon measured 21.0 x 17.5 cm, and the aforementioned 8.0 cm fecalith was seen at the rectosigmoid junction (Figure 2). Given the location of the fecalith, it was believed to be causing a chronic, partial mechanical obstruction, resulting in the creation of the more proximal fecaloma. Gastrointestinal and general surgery teams were consulted for possible removal of the fecalith and fecaloma; however, given that the patient was not completely obstructed and the situation was non-emergent, he was subsequently admitted to internal medicine for non-operative management of constipation.

**Figure 3:**
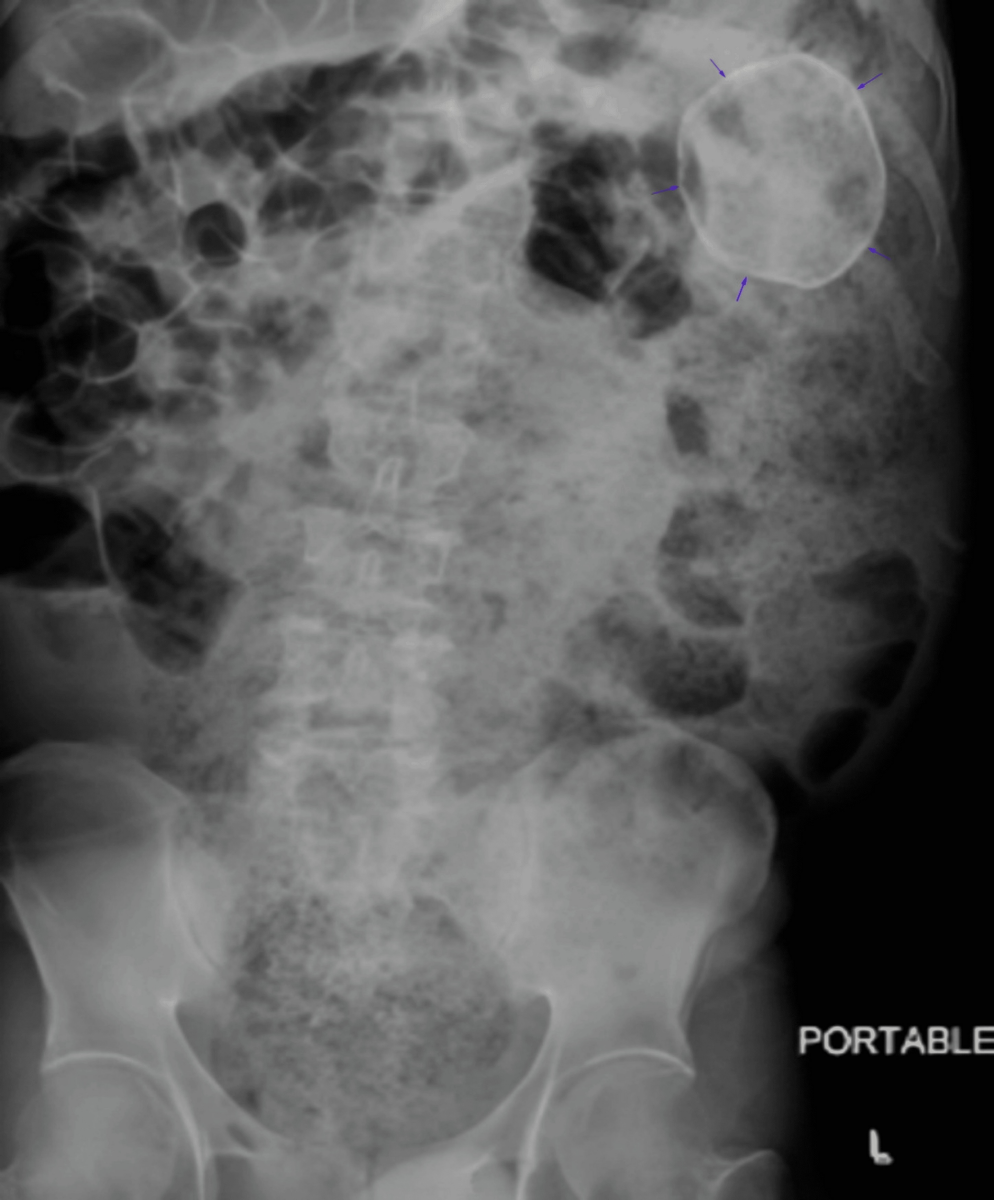
Plain film abdominal X-ray demonstrating movement of the fecalith (arrows) into the proximal descending colon.

After several days of conservative management, the patient was noted to have a larger volume and more frequent bowel movements. He was discharged with an aggressive bowel regimen and return precautions, should complete obstruction occur. Upon short-interval follow-up, repeat abdominal X-rays showed movement of the fecalith into the proximal descending colon (Figure 3). Discussion for elective surgery was held with the healthcare proxy; however, given the patient’s resolution of presenting symptoms, the decision was ultimately made to continue with conservative management.

## Discussion

Patients with IDs have increased healthcare needs that make treatment for common issues more difficult than in the general population. In chronic constipation, meticulous care and diligent follow-up are needed to prevent serious complications in this patient population. Several approaches to the management of constipation in this population are discussed in this report.

Medical management

Consistency in Bowel Regimen

Maintaining a daily bowel regimen in ID patients is the first line, and arguably the most important, therapy in dealing with chronic constipation. Since these patients often rely on the schedules and abilities of individual or group caregivers, they are limited in their physical activity and dietary choices. Using daily polyethylene glycol, and increased fluid and dietary fiber intake, the most common methods of constipation relief in these patients will usually allow for the establishment of a “baseline” bowel evacuation that caregivers can monitor for changes [3,5].

When this population is maintained on a daily bowel regimen, efforts may be initially effective but less so over time. Hence, agreed protocols should be implemented regarding escalation strategies in order to afford better overall patient outcomes and decreased healthcare utilization [10,14]. Additional oral constipation medications, such as senna glycosides, bisacodyl, magnesium hydroxide, and enemas, can be added to maximize the frequency of bowel movements as needed and titrated to produce one to two soft, well-formed bowel movements daily [9,14]. Rectal suppositories can be used in patients with dyssynergic defecation to assist with bowel evacuation. Trans-anal irrigation can be an effective treatment if enemas are ineffective; however, it should be done sparingly, as the application may contribute to rectal mucosal damage. Formulations intended for colonoscopy preparation may also be utilized in those patients in whom a mechanical obstruction has been ruled out [9-11,14].

Long-Term Caregiver Support

Patients with IDs who have access to consistent and individualized caregiver support, or those who live in specialized housing communities for IDs, have been found to have better constipation outcomes than those without constant care [13]. Caregivers and continuity care teams are crucial in assisting with the administration of medications, follow-up visits, and relaying information to physicians that the patient cannot otherwise verbalize. In the setting of chronic constipation, caregivers or group facilities are as much front-line providers as the physician, ensuring the alleviation of symptoms and advocating on behalf of the patients for the best possible outcomes [14]. It is imperative that physicians interact and communicate medical goals with caregivers and community care teams to provide a multi-disciplinary approach that promotes continuity of care outside of the healthcare setting.

Ethics, Goals of Care, and Healthcare Proxy

One of the most common problems that physicians encounter when escalating care for persons with IDs is navigating goals of care with a healthcare proxy. Especially in non-emergent scenarios, physicians oftentimes have difficulty escalating care past conservative measures. This prevents the treatment of the primary cause of constipation and ultimately leads to more hospital and clinic encounters. It is important that these patients have documented long-term goals of care from a physician at their living facility or a designated healthcare proxy (family member/full-time caregiver), who understands the patient’s mannerisms, potential expressed desires, and comfortability [18]. Obtaining official palliative care or ethics consult from a hospital or state agency to explicitly document the appropriate progression of management can also limit unnecessary tests or allow patients to receive non-emergent interventions or surgical procedures, if needed, and therefore improve the overall quality of life in this population [15].

Surgical management

When lifestyle modification and medical management are unsuccessful, surgery becomes an option. In patients who fail nonoperative management, surgical intervention can provide a safe and viable solution [16,17]. When surgical intervention is considered, it should be done with the intent to improve the patient’s quality of life. Surgical intervention can initially be an off-putting proposition to some; however, when you take into account the discomfort caused by severe constipation, the constant need to stay balanced and administer multi-component bowel regimens, the side effects of medications, enemas, and disimpaction, the necessity for frequent visits to the emergency department, and the other numerous resources, including transportation and man-hours, placed on providing symptomatic relief, surgery can be an economically feasible option that is associated with low morbidity [9,15-17].

When surgical intervention is considered, it would be prudent for the patient to undergo a pre-operative colonoscopy. Colonoscopy is usually required prior to any elective surgical resection, as it will help rule out partial obstruction secondary to a neoplastic process, which would ultimately change the surgical plan from a non-formal to an oncological resection. Additionally, in the setting of constipation, a colonoscopy can not only be diagnostic but also therapeutic, as the bowel prep necessary to undergo a colonoscopy has a high rate of bowel evacuation [16,19]. In the uncommon instance that the patient isn’t able to fully evacuate his or her bowels, a dual prep usually accomplishes the goal. Oftentimes, patients will exhibit relief of constipation symptoms during an interval period immediately following the completion of a bowel prep.

For persons such as this patient with extreme, severe constipation, any treatment other than surgery usually has a low success rate. Surgical intervention would be targeted at the believed etiology of constipation and further tailored to address concomitant comorbidities [16,17]. Cross-sectional imaging, such as CT or MRI, colonoscopy, and even defecography, would be obtained preoperatively and play a crucial role in identifying anatomic abnormalities (prolapse, malignancy, stricture, rectocele, etc.), all things that would change the surgical plan [17]. Some important questions to consider when determining the ideal surgical intervention in a patient with IDs and chronic, severe constipation include duration of symptoms, physical/mental capabilities, continence status, available assistance in the home setting, and individual goals [17]. Generally speaking, surgical intervention would focus on one or both of the following approaches: diversion and resection.

Diversion

Significant relief of constipation symptoms can be achieved by diverting stool flow proximal to an obstruction (in this case, the fecaloma), with the creation of an ostomy.

Diversion options would include the creation of a colostomy or ileostomy, and these ostomies would be in the construct of either an end or loop ostomy. Several factors would go into determining which construct would benefit a patient the most, with some considerations being the planned location of creation in the GI tract (how far along in the GI tract), ileocecal valve function, and potential for reversal [17]. With end ostomies, the creation of a new “end of the GI tract” would involve transecting the GI tract proximal to the fecaloma, and only an afferent limb of bowel would exist within the ostomy; the distal limb could be left as a long pouch (Hartmann fashion) or also brought up to the skin to create a mucous fistula. With loop ostomies, the GI tract is left in continuity from the mouth to the anus; however, a portion of the bowel at a designated location somewhere proximal to the fecaloma is brought up to the abdominal wall, and both an afferent and efferent limb would contribute to the construct of the ostomy [16].

Ileostomies and colostomies each have their advantages and disadvantages. Ileostomies have a comparatively low herniation and prolapse rate; however, their output volume can be difficult to control [17]. This can be problematic from a volume status point of view, as it places patients at risk for dehydration, acute kidney injury, and significant metabolic derangement. Considering patients with IDs, who oftentimes have low PO intake at baseline, the risk for dehydration and electrolyte abnormalities is especially high in this population [6,16]. Additionally, due to their more proximal location, as well as the liquid nature and high alkaline character of succus, the surrounding skin is at a much higher risk for breakdown and maceration when compared to a colostomy [17].

A transverse loop colostomy would likely be the best-isolated diversion option for a patient with a large fecaloma in the descending and sigmoid colon, as it would divert stool proximally and provide a venting option for distal gas and mucous. In reality, any loop colostomy proximal to the obstruction would likely succeed in providing the patient with symptomatic relief [16]. It provides a way for waste to be excreted just proximal to the fecaloma, would mitigate the potential for dehydration, and would also allow for antegrade enemas to be performed in an attempt to break down and clear out the fecaloma [16,17].

Resection

Another approach to consider would be resection in the emergency setting due to perforation. With resection, either a subtotal or total colectomy would need to be performed to remove the diseased segment of the colon. Following resection, reconstruction would occur with either a primary anastomosis (with or without a temporary loop ileostomy for anastomotic protection) or be paired with a permanent diversion construct [16]. A primary anastomosis would maintain the continuity of the GI tract, spare the patient from the morbidity of an ostomy, and would be ideal for ID patients who still maintain their continence. In patients who are incontinent, resection paired with diversion would likely be the least morbid and most successful option [17].

## Conclusions

Patients with severe IDs frequently have exacerbations of chronic constipation. Treating constipation in patients with IDs presents a complex and multifaceted challenge. This case highlights the severe sequelae resulting from untreated constipation in individuals with IDs, resulting in fecaloma formation and leading to large bowel obstruction. Additionally, this case highlights the nuances and importance of multi-disciplinary management to effectively recognize and provide proactive management strategies. Individuals with IDs often experience difficulties in effectively communicating their discomfort and pain, making it more challenging to identify and address constipation in its early stages. The cognitive and physical limitations they face can lead to poor dietary choices, limited fluid intake, and reduced physical activity, all of which contribute to worsening constipation. Lifestyle modifications and medical management are often successful in providing symptomatic relief. However, when conservative management fails, surgical intervention should be considered as a last resort after careful discussions with a multi-disciplinary team. Constipation in patients with IDs necessitates a tailored and patient-centered approach that involves caregivers, healthcare providers, and specialists collaborating closely to develop strategies to accommodate the unique needs of each individual, with the overall goal of ensuring adequate relief from constipation while maintaining their overall well-being. Through effective monitoring, recognition, and treatment, we, as a community, can continue to work toward preventing complications, which ultimately will warrant operative intervention in this patient population.
